# Supplementary material for: Does Baseline Hounsfield Unit Predict Patients’ Outcomes Following Surgical Management of Unstable Osteoporotic Thoracolumbar Fractures?
Source: Medicina (Kaunas). 2025 Jan 27;61(2):227. doi: 10.3390/medicina61020227 (PMC11857692; doi:10.3390/medicina61020227)
Supplement: Supplementary file 1 [file medicina-61-00227-s001.zip › medicina-3400155-supplementary.pdf]

Table S1. Multivariate linear regression model showing the predictors of COMI score

	Coefficient	SE	T	P	Low CI	High CI
**HU Quartile [Reference Group: Q1 < 56.24]**						
**Q2: 56.24 - 72.63**	0.433	3.223	0.130	0.896	-6.858	7.723
**Q3: 72.63 - 87.59**	-0.677	2.691	-0.250	0.807	-6.764	5.409
**Q4 >87.59**	-4.473	2.880	-1.550	0.155	-10.989	2.044
**Fracture Level [Reference Group: Lumbar]**						
**Thoracic**	0.009	1.413	0.010	0.995	-3.188	3.207
**Pfirmann Classification [Reference Group: II]**						
**III**	5.255	4.002	1.310	0.222	-3.797	14.307
**IV**	2.715	2.624	1.030	0.328	-3.221	8.652
**V**	3.889	3.138	1.240	0.247	-3.209	10.988
**BMI Category [Reference Group: 20-25 / Normal Weight]**						
**<20**	4.259	4.302	0.990	0.348	-5.473	13.991
**25-30**	-1.173	2.569	-0.460	0.659	-6.984	4.638
**30-35**	1.298	2.837	0.460	0.658	-5.119	7.714
**ASA Category [Reference Group: II]**						
**I**	-2.289	4.272	-0.540	0.605	-11.954	7.375
**III**	0.021	2.869	0.010	0.994	-6.468	6.510
**Constant**	2.405	3.231	0.740	0.476	-4.905	9.715

MISS/LISS therapy was excluded due to significant collinearity with fracture level. Patients' age at death was excluded due to insufficient number of observations. SE: standard error; CI: confidence interval.

Table S2. Multivariate linear regression model showing the predictors of quality of life

	Coefficient	SE	T	P	Low CI	High CI
**HU Quartile [Reference Group: Q1 < 56.24]**						
**Q2: 56.24 - 72.63**	0.411	0.408	1.010	0.340	-0.512	1.334
**Q3: 72.63 - 87.59**	0.324	0.341	0.950	0.367	-0.447	1.094
**Q4 >87.59**	0.447	0.365	1.230	0.251	-0.378	1.272
**Fracture Level [Reference Group: Lumbar]**						
**Thoracic**	0.080	0.179	0.450	0.665	-0.325	0.485
**Pfirmann Classification [Reference Group: II]**						
**III**	0.243	0.507	0.480	0.643	-0.903	1.389
**IV**	-0.063	0.332	-0.190	0.853	-0.815	0.688
**V**	0.047	0.397	0.120	0.908	-0.851	0.946
**BMI Category [Reference Group: 20-25 / Normal Weight]**						
**<20**	0.380	0.545	0.700	0.502	-0.851	1.612
**25-30**	0.387	0.325	1.190	0.264	-0.348	1.123

**30-35**	0.244	0.359	0.680	0.513	-0.568	1.057
**ASA Category [Reference Group: II]**						
**I**	-0.166	0.541	-0.310	0.766	-1.389	1.058
**III**	-0.413	0.363	-1.140	0.284	-1.235	0.408
**Constant**	0.146	0.409	0.360	0.729	-0.779	1.072

MISS/LISS therapy was excluded due to significant collinearity with fracture level. Patients' age at death was excluded due to insufficient number of observations. SE: standard error; CI: confidence interval

Table S3. Multivariate linear regression model showing the predictors of back pain score

	**Coefficient**	**SE**	**T**	**P**	**Low CI**	**High CI**
**HU Quartile [Reference Group: Q1 < 56.24]**						
**Q2: 56.24 - 72.63**	-0.842	3.038	-0.280	0.788	-7.716	6.031
**Q3: 72.63 - 87.59**	-2.108	2.537	-0.830	0.427	-7.846	3.630
**Q4 >87.59**	-3.409	2.716	-1.260	0.241	-9.553	2.734
**Fracture Level [Reference Group: Lumbar]**						
**Thoracic**	0.050	1.333	0.040	0.971	-2.964	3.065
**Pfirmann Classification [Reference Group: II]**						
**III**	1.484	3.773	0.390	0.703	-7.050	10.018
**IV**	-0.144	2.474	-0.060	0.955	-5.741	5.453
**V**	4.086	2.958	1.380	0.201	-2.606	10.779
**BMI Category [Reference Group: 20-25 / Normal Weight]**						
**<20**	1.426	4.056	0.350	0.733	-7.749	10.601
**25-30**	-0.180	2.422	-0.070	0.942	-5.659	5.298
**30-35**	1.648	2.674	0.620	0.553	-4.402	7.697
**ASA Category [Reference Group: II]**						
**I**	0.346	4.028	0.090	0.933	-8.765	9.458
**III**	-0.118	2.704	-0.040	0.966	-6.236	6.000
**Constant**	3.770	3.047	1.240	0.247	-3.121	10.662

MISS/LISS therapy was excluded due to significant collinearity with fracture level. Patients' age at death was excluded due to insufficient number of observations. SE: standard error; CI: confidence interval

Table S4. Multivariate linear regression model showing the predictors of alpha angle

	**Coefficient**	**SE**	**T**	**P**	**Low CI**	**High CI**
**HU Quartile [Reference Group: Q1 < 56.24]**						
**Q2: 56.24 - 72.63**	5.204	5.625	0.930	0.379	-7.520	17.928
**Q3: 72.63 - 87.59**	6.187	4.696	1.320	0.220	-4.436	16.809
**Q4 >87.59**	3.324	5.027	0.660	0.525	-8.049	14.696
**Fracture Level [Reference Group: Lumbar]**						

**Thoracic**	1.204	2.467	0.490	0.637	-4.377	6.784
**Pfirmann Classification [Reference Group: II]**						
**III**	8.759	6.984	1.250	0.241	-7.040	24.557
**IV**	1.157	4.580	0.250	0.806	-9.204	11.519
**V**	1.939	5.477	0.350	0.731	-10.450	14.329
**BMI Category [Reference Group: 20-25 / Normal Weight]**						
**<20**	4.466	7.509	0.590	0.567	-12.519	21.452
**25-30**	-1.917	4.483	-0.430	0.679	-12.059	8.226
**30-35**	0.411	4.951	0.080	0.936	-10.789	11.610
**ASA Category [Reference Group: II]**						
**I**	2.401	7.456	0.320	0.755	-14.467	19.268
**III**	-7.598	5.007	-1.520	0.163	-18.924	3.728
**Constant**	7.841	5.640	1.390	0.198	-4.917	20.600

MISS/LISS therapy was excluded due to significant collinearity with fracture level. Patients' age at death was excluded due to insufficient number of observations. SE: standard error; CI: confidence interval

Table S5. Multivariate logistic regression model showing the predictors of overall complications

	aOR	SE	T	P	Low CI	High CI
**HU Quartile [Reference Group: Q1 < 56.24]**						
**Q2: 56.24 - 72.63**	1.051	1.013	0.050	0.959	0.159	6.953
**Q3: 72.63 - 87.59**	0.907	0.778	-0.110	0.909	0.169	4.877
**Q4 >87.59**	0.162	0.171	-1.720	0.085	0.021	1.283
**Fracture Level [Reference Group: Lumbar]**						
**Thoracic**	0.815	0.586	-0.280	0.776	0.199	3.339
**Thoracolumbar**	1.000	(empty)				
**Pfirmann Classification [Reference Group: II]**						
**III**	1.240	1.125	0.240	0.813	0.209	7.340
**IV**	0.619	0.578	-0.510	0.608	0.099	3.864
**V**	4.131	5.263	1.110	0.266	0.340	50.184
**BMI Category [Reference Group: 20-25 / Normal Weight]**						
**<20**	0.162	0.297	-0.990	0.320	0.005	5.850
**25-30**	0.282	0.230	-1.550	0.121	0.057	1.399
**30-35**	0.179	0.173	-1.780	0.075	0.027	1.193
**35-40**	1.000	(empty)				
**ASA Category [Reference Group: II]**						
**I**	1.000	(empty)				
**III**	0.709	0.533	-0.460	0.647	0.163	3.092
**IV**	1.000	(empty)				
**Constant**	3.966	4.277	1.280	0.201	0.479	32.827

MISS/LISS therapy was excluded due to significant collinearity with fracture level. Patients' age at death was excluded due to insufficient number of observations. aOR: adjusted odds ratio; SE: standard error; CI: confidence interval.
